# VEXAS syndrome in rheumatology practice: features from a multicenter cohort in north-east Italy

**DOI:** 10.3389/fimmu.2025.1700737

**Published:** 2025-12-02

**Authors:** Sara Bindoli, Greta Morello-Pasin, Irina Guidea, Roberto Padoan, Luca Iorio, Riccardo Bixio, Giovanni Orsolini, Federica Maiolini, Sara Lombardi, Sandra Lombardi, Marta Favero, Cristina Bernardi, Bernd Raffeiner, Andrea Doria, Roberta Ramonda, Paolo Sfriso

**Affiliations:** 1 Rheumatology Unit, Azienda Ospedale Università di Padova, Padova, Italy; 2 Department of Medicine (DIMED), Università degli Studi di Padova, Padova, Italy; 3 Unit of Rheumatology, IRCCS Sacro Cuore Don Calabria Hospital, Verona, Italy; 4 Rheumatology Unit, Department of Medicine, Università degli Studi di Verona, Verona, Italy; 5 Unit of Internal Medicine B, Department of Medicine, Università degli Studi di Verona, Verona, Italy; 6 General Medicine Unit, Girolamo Fracastoro Hospital, AULSS9 Scaligera, Verona, Italy; 7 Struttura Complessa Medicina Interna, Gorizia Hospital, Gorizia, Italy; 8 Internal Medicine 1, ULSS2 Marca Trevigiana, Ca’ Foncello Hospital, Treviso, Italy; 9 Rheumatology Unit, San Giovanni e Paolo Hospital, Venice, Italy; 10 Department of Rheumatology, Teaching Hospital of the Paracelsius Medical Hospital of Bolzano (ASAA-SABES), Bolzano, Italy

**Keywords:** VEXAS syndrome, inflammation, chondritis, hematologic abnormalities, genetics

## Abstract

**Objective:**

VEXAS syndrome (Vacuoles, E1 enzyme, X-linked, Autoinflammatory, Somatic) is a late-onset autoinflammatory disorder caused by somatic mutations in the *UBA1* gene. It is characterized by systemic inflammation, a wide spectrum of rheumatologic features, including chondritis and inflammatory arthritis, dermatologic manifestations (e.g. neutrophilic dermatosis or vasculitis-like lesions), and hematologic abnormalities like macrocytic anemia and myelodysplastic syndrome. Due to its heterogeneity, diagnosis is frequently delayed. Early recognition of hallmark inflammatory symptoms, particularly by rheumatologists, is critical for timely diagnosis and management.

**Methods:**

We conducted a retrospective analysis of 37 patients over the age of 50. Next-Generation Sequencing (Illumina HiSeq2500) was employed to assess mutations. Clinical, genetic, and demographic data were extracted from electronic medical records.

**Results:**

Twenty patients [100% male; median age 73 years (IQR 67–77)] were confirmed to carry somatic *UBA1* mutations. All patients exhibited constitutional symptoms (100%) and at least one rheumatologic manifestation, including chondritis (75%), arthralgia or arthromyalgia (50%), arthritis (30%), osteopenia or osteoporosis (15%), myalgia or myositis (10%), and tenosynovitis (5%). Dermatologic and hematologic abnormalities frequently co-occurred. Infectious complications were observed in 80% of patients and were a major contributor to overall morbidity.

**Conclusion:**

This study underscores the need for a phenotype-driven diagnostic approach to facilitate earlier identification of VEXAS syndrome. Our findings suggest that current estimates of prevalence in rheumatology settings may significantly underestimate the true disease burden. Improved awareness and interdisciplinary collaboration, particularly among rheumatologists, hematologists, and dermatologists, are essential to enhance recognition, diagnosis, and comprehensive care for individuals affected by this complex syndrome.

## Introduction

VEXAS syndrome (Vacuoles, E1 enzyme, X-linked, Autoinflammatory, Somatic) is an adult-onset autoinflammatory disorder caused by somatic loss-of-function mutations in the *UBA1* gene within hematopoietic progenitor cells (1, 2). These mutations disrupt the function of the E1 ubiquitin-activating enzyme, leading to impaired protein degradation pathways and a broad, heterogeneous clinical phenotype. Systemic symptoms frequently include fever, night sweats, and unintentional weight loss, while organ-specific involvement most commonly affects the dermatologic and rheumatologic domains. Hematologic abnormalities, particularly those affecting the myeloid lineage, are also prevalent, with macrocytic anemia and features compatible with myelodysplastic syndrome (MDS) being the most frequently reported.

A recent systematic review (SR) by Al-Hakim et al. (3) analyzed 720 patients across 33 case reports and 21 case series. This SR confirmed that skin involvement is the most common clinical feature observed, affecting 81.8% of patients (95% CI: 78.8–84.5%), followed by constitutional symptoms in 69.4% (95% CI: 66.0–72.7%), and respiratory manifestations in 61.3% (95% CI: 57.6–64.7%). Joint involvement was instead reported in 47.3% of patients (95% CI: 43.5–51.2%), ocular inflammation in 44.3% (95% CI: 40.5–48.2%), and venous thromboembolism in 41.8% (95% CI: 38.3–45.4%). In contrast, MDS was observed in 35.8% of cases (95% CI: 32.3–39.4%), suggesting that the recurrence of inflammatory and autoimmune-like manifestations often outweighs the prominence of hematologic findings in initial clinical presentation.

Given the predominance of rheumatologic and dermatologic features, many of which mimic known autoimmune and autoinflammatory conditions, rheumatologists are likely to encounter VEXAS patients within their routine clinical practice.

The objective of this study is to describe the recurrence and distribution of key inflammatory clinical features of VEXAS syndrome, primarily as observed in rheumatological settings, to raise awareness among rheumatologists.

To this end, we collected data from patients with a high clinical suspicion of VEXAS syndrome who were referred to multiple rheumatology and internal medicine centers across the TriVeneto macro-region in northeastern Italy. This area, which has a catchment population of approximately 7,129,534 inhabitants, includes an estimated 3,386,000 individuals over the age of 50. Notably, around 1.5 million of them are males, representing 44% of this age group, and constitute the primary demographic at risk for this syndrome (4).

## Patients and methods

Patients exhibiting clinical manifestations and laboratory findings consistent with a presumptive diagnosis of VEXAS syndrome were recruited from hub-and-spoke Hospitals and Outpatient Clinics across the Veneto, Trentino-Alto Adige, and Friuli Venezia Giulia Regions. The diagnosis of VEXAS syndrome was based on the identification of *UBA1* mutations via Next-Generation Sequencing (NGS) (*HiSeq2500 Illumina Sequencer*) and demographic data were extracted from electronic medical records. Disease activity was retrospectively assessed using the recently developed VEXAS Disease Activity Index (VEXAS-DAI), based on clinical data documented at symptom’s onset (5). This score includes 12 scored domains, each with a specified number of items: inflammatory-type rash (2), chondritis (3), ophthalmologic involvement (5), periorbital involvement (1), joint (1), pulmonary (3), cardiovascular (3), genitourinary (1), neurologic (5), oral and gastrointestinal (3), renal (1), and constitutional symptoms (present or absent). Additionally, thrombosis and thromboembolism domain is included but remains unscored. Scores range from 0 to 40.

All participants provided written informed consent prior to inclusion in the study, which was conducted in accordance with the principles of the Declaration of Helsinki. The study protocol received approval by the Ethics Committee of Padova University Hospital (protocol code 5349/AO/22).

### Statistical analysis

The distribution of continuous variables was assessed using the Shapiro-Wilk test. Variables that did not follow a normal distribution were reported as medians with their corresponding IQRs. Correlations between variables were evaluated using Spearman’s rank correlation coefficient. All statistical analyses were conducted using GraphPad Prism version 10 (*GraphPad Software Inc., La Jolla*), with a significance threshold set at p < 0.05.

## Results

### Clinical features

A total of 37 patients (100%) were recruited. Of these, 20 patients (54%), all male, with a median age of 73 years (IQR 67–77), were diagnosed with VEXAS syndrome based on the identification of *UBA1* mutations through next-generation sequencing (NGS). Genetic variants, along with their respective variant allele fractions (VAF%), clinical manifestations, prior diagnoses, diagnostic delays, and current treatment regimens are summarized in **Table 1**, while the rheumatologic manifestations observed are summarized in **Table 2**. All patients exhibited at least one rheumatologic feature during the disease course. Constitutional symptoms, including asthenia, fatigue and weight loss, were reported in all the patients (100%), and 15 individuals (75%) experienced recurrent febrile episodes.

**Table 1:** Demographic and genetic aspects along with previous diagnosis and diagnostic delay time of patients with VEXAS syndrome.

ID	Age (years)	*UBA1* mutation	VAF	Total VEXAS-DAI	Diagnostic delay (years)	Previous diagnosis	Therapy undertaken after diagnosis
P01	68	c.121A>C (p.Met41Leu)	90%	12.3	3	Cutaneous Vasculitis	Methyl-prednisolone, Filgotinib
P02	62	c.122C>T (p.Met41Thr)	NA	10	2	Sweet Syndrome/Polyarteritis nodosa	Prednisone, Upadacitinib (discontinued for major infection)
P03	84	c.121A>C (p.Met41Leu)	65%	8.93	2	Chronic recurrent urticaria	Prednisone, Colchicine
P04	87	c.118-1G>C	51.3%	3	3	Crystal-induced arthritis, polymyalgia	Prednisone, Filgotinib (discontinued for major infection)
P05	82	c.121A>G (p.Met41Val)	62.5%	8.8	1	Undifferentiated AID/GCA	Prednisone, Methotrexate
P06	73	c.122C>T (p.Met41Thr)	81%	6.13	5	Vasculitis (Schoenlein-Henoch purpura)	Prednisone
P07	87	c.122C>T (p.Met41Thr)	63.2%	6	NA	FUO	Prednisone
P08	72	c.118-2A>G	74.6%	8.13	6	Relapsing Polychondritis	Prednisone, Methotrexate, Filgotinib (both discontinued for major infection)
P09	77	c.121A>G (p.Met41Val)	32.5%	3.8	NA	Undifferentiated AID	Prednisone
P10	75	c.122C>T (p.Met41Thr)	59.1%	5.13	NA	Relapsing Polychondritis	Prednisone
P11	72	c.122C>T (p.Met41Thr)	72.2%	6.8	4	Seronegative arthritis	Prednisone, Tocilizumab (discontinued for major infection)
P12	78	c.122C>T (p.Met41Thr)	60%	4.8	7	Relapsing Polychondritis	Prednisone, Filgotinib,
P13	73	c.122C>T (p.Met41Thr)	NA	4.8	NA	Relapsing Polychondritis	Prednisone, Canakinumab
P14	60	c.122C>T (p.Met41Thr)	15%	3.4	NA	CTD	Prednisone, Methotrexate
P15	65	c.118-1G>C	NA	8.66	NA	Behçet Disease	Prednisone
P16	52	c.122C>T (p.Met41Thr)	NA	3	NA	IgG4-RD	Prednisone, Ruxolitinib (discontinued for major infection)
P17	77	c.121A>G (p.Met41Val)	53.4%	9.66	2	Undifferentiated AID	Prednisone, Ruxolitinib, (previously Tocilizumab)
P18	72	c.121A>G (p.Met41Val)	27.8%	4.8	NA	Undifferentiated AID	Prednisone
P19	57	c.121A>G (p.Met41Val)	47%	6	NA	Undifferentiated AID	Prednisone, Azacytidine
P20	68	c.121A>G (p.Met41Val)	20.3%	4.13	7	Behçet Disease	Prednisone

AID, autoinflammatory Disease; CTD, connective tissue disease; DAI, disease activity index; FUO, fever of unknown origin; GCA, Giant Cell Arteritis; NA, not available; RD, related disease.

**Table 2:** Clinical symptoms exhibited by the patients subdivided by domain.

Clinical features	N(%)
Musculo-skeletal features	16/20 (80%)
*Arthralgia/arthromyalgias* *Arthritis* *Osteoporosis* *Osteopenia* *Tenosynovitis*	10/20 (50%) 6/20 (30%) 2/20 (10%) 1/20 (5%) 1/20 (5%)
Dermatologic features	16/20 (80%)
*Vasculitis-like lesions (including purpura, livedo reticularis and leukocytoclastic vasculitis)* *Erythema nodosum/EN-like lesions* *Cutaneous nodules* *Maculopapular rash* *Sweet syndrome* *Neutrophilic dermatosis* *Non-defined rash* *Fasciitis*	6/16 (37.5%) 4/16 (25%) 3/16 (18.7%) 3/16 (18.7%) 2/16 (12.5%) 1/16 (6.2%) 1/16 (6.2%) 1/16 (6.2%)
*Chondritis*	15/20 (75%)
Isolated nasal chondritis Isolated auricular chondritis Both nasal and auricular chondritis	0 6/15 (40%) 9/15 (60%)
Ocular involvement	11/20 (55%)
Scleritis/episcleritis Orbital cellulitis Orbital oedema	6/11 (54.5%) 3/11 (27.2%) 2/11 (18.2%)
Lung Involvement	6/20 (30%)
Pleuritis ILD DAH	5/6 (83.3%) 1/6 (16.6%) 1/6 (16.6%)
Central Nervous System	
*Peripheral neuropathy* *Central nervous system*	3/20 (15%) 0
*Orchitis*	3/20 (15%)
Constitutional symptoms	20/20 (100%)
Fever Asthenia, fatigue, weight loss	15/20 (75%) 20/20 (100%)
Thrombotic events	14/20 (70%)
Isolated DVT Concurrent SVT Isolated SVT	9/14 (64.2%) 3/14 (21.4%) 2/14 (14.2%)
Hematological abnormalities	20/20 (100%)
• *Anemia* Macrocytic Normocytic	19/20 (95%) 17/19 (89.4%) 2/19 (10.5%)
• *Thrombocytopenia*	2/20 (10%)
• *MDS*	10/20 (50%)
• *MGUS*	2/20 (10%)
• *JAK2 V617F mutation*	1/20 (5%)

DAH, diffuse alveolar hemorrhage; DVT, deep vein thrombosis; EN, erythema nodosum; ILD, interstitial lung disease; MDS, myelodysplastic syndrome; MGUS, monoclonal gammopathy of undetermined significance; SVT, superficial vein thrombosis.

**Figure 1:**
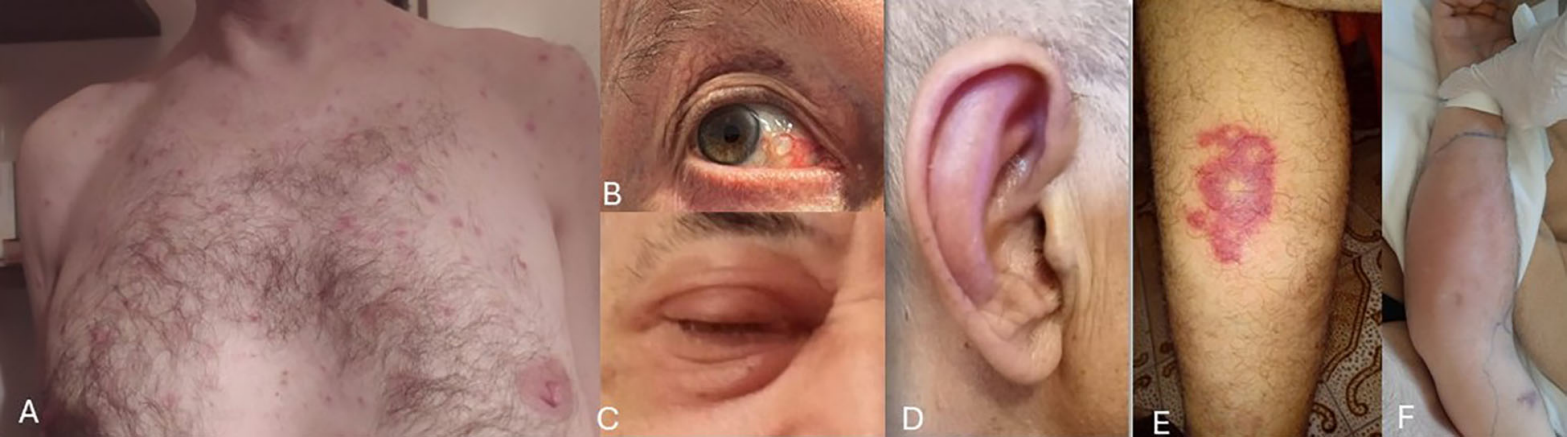
Images depicting selected clinical features affecting the skin and eyes in our VEXAS patients (with patients’ permission). **(A)** Truncal neutrophilic dermatosis; **(B)** Ocular scleritis; **(C)** Periorbital oedema; **(D)** Ear chondritis; **(E)** Vasculitic lesion on lower limbs; **(F)** Arm fasciitis with marked subcutaneous oedema.

Arthritis was diagnosed in 6 out of 20 patients (30%), while arthralgia and/or myalgia were observed in 10 out of 20 cases (50%). Chondritis was present in 15/20 patients (75%); of these, 6/15 (40%) had isolated auricular involvement, and the remaining 9/15 (60%) presented with both auricular and nasal chondritis (**Figure 1D**). Notably, no cases of isolated nasal chondritis were identified. Tenosynovitis was reported in one patient (5%).

Regarding bone metabolism, reduced bone mineral density was documented in a minority of patients. Overall, one individual (5%) had osteopenia, and two out of 20 patients (10%) had osteoporosis, one of whom also experienced vertebral fractures.

Cutaneous manifestations (**Figures 1A, E, F**) were frequent and heterogeneous, occurring overall in 16 out of 20 patients (80%). The detailed summary is provided in **Table 2**. Neutrophilic dermatosis, confirmed histologically by neutrophilic infiltration, was identified only in one out of 16 patients (6.2%). Sweet’s syndrome was diagnosed in 2/16 patients (12.5%), maculo-papular rash and cutaneous nodules in 3/16 patients (18.75%), respectively. Vasculitis-like lesions instead were observed in 6/16 patients (37.5%) and these included purpuric rashes, livedo reticularis and histologically confirmed leukocytoclastic vasculitis. Erythema nodosum (EN) or EN-like lesions occurred in 4/16 cases (25%). Additional dermatologic features included a non-specified rash in 1 patient out of 16 (6.2%), and bilateral arm fasciitis with significant subcutaneous oedema and diffuse redness (1 patient, 6.2%).

Although ocular involvement is not primarily managed within rheumatological practice, it is well represented in several autoinflammatory and autoimmune conditions. Indeed, also in our cohort, ocular manifestations were prominent, affecting 55% of patients (11/20). Among these eleven subjects, the majority presented with scleritis/episcleritis (54.5%), followed by orbital cellulitis (27.2%) and orbital edema (18.2%) (**Figures 1B, C**).

**Figure 2:**
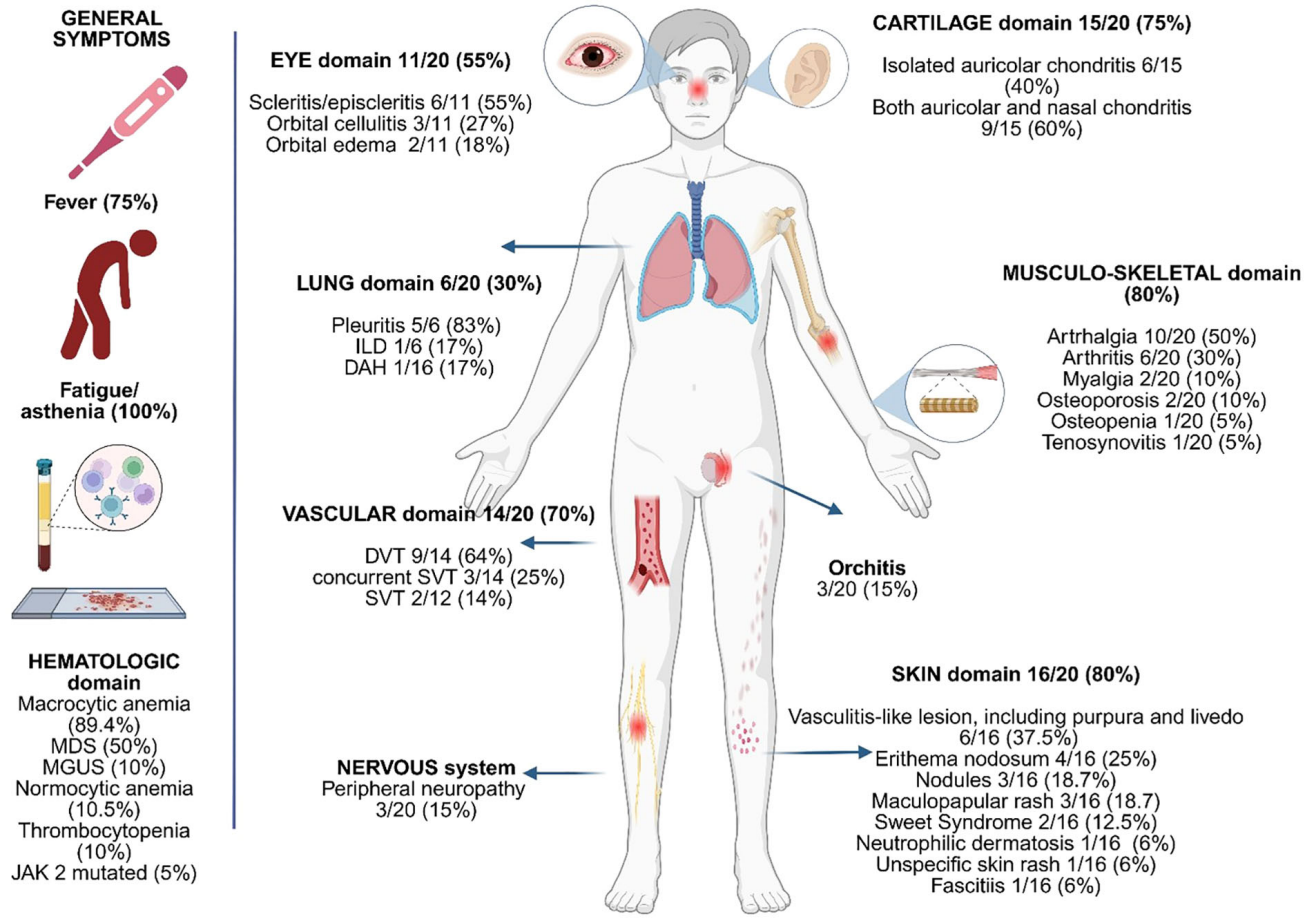
Graphical presentation of the clinical and biological features of the patients included.

Among the non-rheumatologic features, anemia was the most consistent manifestation, observed in 19 out of 20 patients (95%), with the macrocytic form present in 89.4% of the cases, reinforcing its role as a key, although non-specific, diagnostic marker in VEXAS syndrome. Myelodysplastic syndrome (MDS) was present in 50% of cases, highlighting the pathogenic association between *UBA1* somatic mutations and clonal hematopoiesis. One patient was found to carry the JAK2 Val617Phe (V617F) mutation. The presence of both macrocytic anemia and MDS, especially within a context of systemic inflammation, should be considered as strongly associated with a possible diagnosis of VEXAS syndrome. Thrombotic events were also common, occurring in 70% of the cohort (14 out of 20 subjects), a frequency higher than previously reported in the literature. **Figure 2** presents a graphical summary of the clinical and biological characteristics observed in our patients.

The VEXAS-DAI score, based on clinical parameters, yielded a mean score of 6.4 and showed a statistically significant positive correlation with the variant allele fraction (r = 0.73, p = 0.0016). Both the individual patients’ VAF values and VEXAS-DAI scores are reported in **Table 1**.

### Infections during the disease course

Of the 20 patients in our cohort, 16 (80%) experienced at least one clinically significant infection, defined as an infection requiring appropriate treatment either in the hospital or outpatient setting during follow-up. In total, 34 infections were documented (**Table 3**). Six patients (37.5%) had a single infectious episode, whereas 10 (62.5%) experienced multiple events throughout the disease course.

**Table 3:** Type of infection developed and ongoing treatment at infections.

	N = 16 (100%)
Treatment at infection	
*Oral glucocorticoids*	16/16 (100%)
*JAK-inhibitors*	4/16 (25%)
*Colchicine*	3/16 (18.7%)
*Methotrexate*	2/16 (12.5%)
*IL-1 inhibitors*	1/16 (6.3%)
Type of event	
*Pneumonia (viral or bacterial)*	10/16 (62.5%)
*(of which fatal legionellosis)*	3/10 (30%)
*SARS-Cov2 infection*	5/16 (31.3%)
*Sepsis*	4/16 (25%)
*Varicella-Zoster re-activation/infection*	3/16 (18.7%)
*Esophageal/oral candidiasis*	3/16 (18.7%)
*Acute bronchitis*	1/16 (6.3%)
*HBV re-activation*	1/16 (6.3%)
*EBV re-activation*	1/16 (6.3%)
*Deep cervicofacial phlegmon*	1/16 (6.3%)
*Bacterial endocarditis*	1/16 (6.3%)
*Severe gastroenteritis with peritonitis*	1/16 (6.3%)
*Influenza A respiratory infection*	1/16 (6.3%)
*Periprosthetic hip bacterial osteomyelitis (Listeria)*	1/16 (6.3%)
*Miliary TBC*	1/16 (6.3%)
*Viral encephalitis*	1/16 (6.3%)
Outcomes	
*Recovery*	13/16 (81.3%)
*Infection-related deaths*	3/16 (18.7%)

The most frequently reported infections involved the respiratory tract, with pneumonia documented in 10 episodes. Among these, 3 cases were caused by *Legionella pneumophila* and resulted in fatal outcomes. Additionally, SARS-CoV-2 infection was noted in 5 episodes. Four episodes of sepsis requiring hospitalization were also observed. Other infections included herpes zoster reactivation (3 episodes) and esophageal/oral candidiasis (3 episodes). Singular events included herpes zoster virus-associated encephalitis, deep cervicofacial phlegmon, severe gastroenteritis with peritoneal signs, bacterial endocarditis, osteomyelitis, acute bronchitis, Epstein-Barr virus (EBV) infection, miliary tuberculosis, and influenza A (H1N1). Clinical outcomes were generally favorable in some cases, with 13 patients (81.3%) experiencing recovery. However, 3 patients (18.7%) died either directly or indirectly as a result of the infection. All patients (100%) were receiving oral corticosteroids at the time of the infectious episode. Additionally, some were undergoing other immunosuppressive therapies: colchicine (3 patients, 18.7%), JAK inhibitors (4 patients, 25%), methotrexate (2 patients, 12.5%), and IL-1 inhibitor canakinumab (1 patient, 6.3%).

## Discussion

VEXAS syndrome is a complex, multisystem disorder characterized by a broad range of manifestations affecting rheumatologic, dermatologic, and hematologic domains. Given its diverse clinical presentation and the requirement for genetic confirmation, diagnosis is often delayed or overlooked, especially in the early disease stages.

In the initial description by Beck et al. (1) dermatologic manifestations were prominent, present in 88% of patients, with nodules being most frequent (52%). Lung involvement was detected in approximately 72%, followed by arthralgia (68%) and chondritis (64%). Similarly, the French cohort reported by Georgin-Lavialle et al. (6) confirmed cutaneous manifestations in 83% of patients, although observed lower frequencies of chondritis (36.2%) and musculoskeletal involvement (28.4%), as well as pulmonary infiltrates (40%). Ocular involvement, however, was relatively frequent, affecting around 40% of patients.

In the Swiss cohort (7), ocular and pulmonary manifestations were among the most common inflammatory features. Cutaneous involvement was again highly prevalent (86%), and musculoskeletal involvement was seen in 47%, with inflammatory arthritis being present in 35% of the patients, whereas chondritis was present in only 24% of the 17 patients included.

A recent Spanish multicenter study (8) involving 39 patients reaffirmed that cutaneous involvement was the most frequent manifestation recorded (87.2%). Notably, articular involvement in this cohort was more prevalent than in others, with polyarthritis reported in 79.4% of patients. This higher rate likely reflects the study’s rheumatologic setting, where many patients had previously received alternate diagnoses such as seronegative arthritis, relapsing polychondritis, polymyalgia rheumatica, Sweet’s syndrome, systemic lupus erythematosus, or medium-vessel vasculitis.

Across cohorts, musculoskeletal involvement showed considerable variability, ranging from 28.3% in the study by Georgin-Lavialle (6) to the nearly 80% of the Spanish cohort (8). The largest SR available (3), including 720 patients, estimated the prevalence of joint involvement at 47.3%, indicating that while it is relatively common, it is not among the most predominant features. In contrast, in the same review skin involvement (81.8%), followed by constitutional symptoms (69.4%), and respiratory manifestations (61.3%) were the most prevalent reported clinical features.

In our cohort, the predominant clinical features aligned with those typically observed in rheumatologic practice. Indeed, musculoskeletal involvement was observed in 80% of patients, with arthralgia in 50% and arthritis in 30%. Chondritis (auricular and/or nasal) and skin manifestations were particularly prevalent, affecting 75% and 80% of the patients, respectively.

Cutaneous manifestations primarily resembled vasculitis-like lesions, observed in 37.5% of patients, with palpable purpura and, more broadly, features consistent with leukocytoclastic vasculitis. EN and EN-like lesions were the next most observed. This pattern contrasts with reports from other cohorts, where maculopapular rashes, papules, and nodules were more often reported (9). Zakine et al. indeed, identified maculopapular eruptions, neutrophilic dermatosis, and arcuate plaques as key cutaneous manifestations, noting that arcuate lesions may be pathognomonic due to their high prevalence (9). In contrast, in the cohort described by Sullivan et al., 19% of the 89 patients demonstrated histopathologic evidence of small-vessel vasculitis, with most exhibiting leukocytoclastic vasculitis on skin biopsy (10). These findings suggest that, while arcuate lesions, plaques, and neutrophilic dermatosis remain distinguishing cutaneous features of VEXAS syndrome, the presence of vasculitic skin lesions should likewise prompt clinically suspicion for VEXAS syndrome. Nevertheless, such manifestations should be interpreted within the broader context of the systemic inflammatory nature of the disease.

The frequency of fever and constitutional symptoms varies considerably across cohorts, with non-infectious fever ranging from 55% (10) to 92% (1), and constitutional symptoms from 46% (10) up to 100%, as observed in our cohort. These manifestations are often self-reported, making precise estimation challenging, particularly given their nonspecific nature. Moreover, such symptoms are commonly observed across a wide spectrum of rheumatic diseases, both autoimmune and autoinflammatory. In this context, it is important to note that patients with VEXAS syndrome are typically older and often burdened with multiple comorbidities, including marked anemia, which may further contribute to the variability and nonspecific nature of these systemic symptoms.

Careful attention should also be given to pulmonary and ocular involvement, both of which are commonly observed in rheumatologic diseases. Respiratory features such as interstitial lung disease (ILD), pleural effusions, pulmonary consolidations, and nodules have been increasingly recognized in VEXAS syndrome (11). Al-Hakim et al. reported that lung involvement constitutes nearly half of all manifestations (3). In our cohort, pulmonary involvement was identified in 30%, with pleuritis being the most frequently feature reported.

Ocular involvement, well documented in VEXAS patients since the earliest reports (12), was observed in up to 59% of patients in the cohort by Wolff et al. (7). In our study, 50% of patients exhibited ocular symptoms, with scleritis/episcleritis being the most common.

Given the frequency of ocular and pulmonary inflammation in VEXAS syndrome, which are features commonly shared with other autoimmune/autoinflammatory conditions, a high index of suspicion is essential, particularly in those presenting with manifestations suggestive of vasculitis, relapsing polychondritis, Behçet’s disease, or even inflammatory arthropathies. These observations underscore the clinical heterogeneity of the disease and highlight the pivotal role of rheumatologists in its early recognition. Supporting this, a monocentric study by Muratore et al. (13) involving 147 patients with confirmed vasculitis, employed targeted *UBA1* mutation screening in those presenting with overlapping rheumatologic, dermatologic, and hematologic features. This approach led to the identification of *UBA1* mutations in three patients on the whole cohort, emphasizing the utility of a phenotype-driven strategy for detecting VEXAS syndrome within rheumatology practice.

Finally, although deep vein thrombosis (DVT) and superficial vein thrombosis (SVT) are not typically considered primary rheumatological manifestations, we observed a notably high incidence of thrombotic events within our cohort. While comprehensive studies on the genetic factors associated with thrombotic risk in VEXAS syndrome remain scarce, we wish to highlight the following findings: one patient tested positive for lupus anticoagulant (LAC) and anti-cardiolipin IgG at 140 GPL/mL (with negative results for IgM and anti-Beta2GP1); another patient was found to be a carrier of HLA-B51, for whom Behçet’s disease had initially been suspected; and a third patient presented with both a mutation in *UBA1* and a mutation in *CECR1* (or *ADA2*), a gene associated with DADA2 syndrome. These observations suggest the need for further investigation into the potential genetic underpinnings of thrombotic events in VEXAS syndrome, extending beyond the well-established mechanism of ‘thrombo-inflammation’ directly driven by the disease itself.

The comparison of the aforementioned cohorts, comprising more than 20 subjects with VEXAS syndrome observed within rheumatological contexts, is summarized in **Table 4**.

**Table 4:** Summary and comparison of clinical characteristics from VEXAS cohorts with over 20 patients.

Clinical and demographic features	*Our cohort (N = 20)*	*Beck et al., 2020 (N = 25)*	*Georgin-Lavialle et al., 2022 (N = 116)*	*Garcia-Escudero et al., 2025 (N = 39)*	*Wolff et al., 2025 (n=23)*	*Sullivan et al., 2025 (n=89)*	*Al-Hakim et al., 2025 (n=720)*
Gender							
Male	100%	100%	96%	100%	100%	100%	98.6%
Female	–	–	4%	–	–	–	1.4%
Age (years), median IQR	73 (67-77)	64 (45–80)	67 (62.5-73)	72.8 (40-92)	74 (59–77)	66.9 (60-73)	NR
Fever	75%	92%	64.7%	79.4%	64%	55%	62%
General symptoms	100%	100% fatigue; 72 % night sweats; 56% wight loss	54.5%	NR	82%	46% weight loss; 20% night sweats	82% (53% fatigue, 44% weight loss,35% night sweats)
*Musculo-skeletal involvement overall*	80%					NR	
Arthralgia/arthromyalgia	50%	68%	28.4%	Not reported	47%		47%
Arthritis	30%	40%	NR	79.4%	35% (SJ 41%; LJ 47%; axial 6%)		34%
Tenosynovitis	5%	NR	NR	NR			NR
Myositis	NR	NR	NR	NR	NR		5%
Myalgia	NR	NR	NR	NR	NR NR		13%
Chondritis	75% (40% auricular, 60% auricular and nasal)	64% (64% auricular, 48% nasal)	36.2% (32% auricular, 15% nasal)	51.2% auricular, 15.3% nasal	24% (12% auricular, 12% costal/tracheal)	38.2%	39% (32% auricular, 12% nasal,3% tracheal, 3% costal)
*Hematologic manifestations*							
Macrocytic anaemia	89.4%	100%	NR	92.3%	100%	85.4%	49%
MDS	50%	36%	50%	46%	71%t	11.2%	11%
MGUS	10%	NR	NR	25.6%	18%	NR	NR
*Dermatologic manifestations overall*	80%	88%	83.6%	87%	86%	NR	82%
Neutrophilic dermatosis	6.2%	NR	39.7%	56.4%	29%	NR	27%
Cutaneous vasculitis	37.5% of which 27% purpura		26%	25.6% (leukocytoclastic vasculitis)	29% (leukocytoclastic vasculitis)	NR	18% 6% livedo racemosa
Erythema nodosum/EN-like lesions	25%		12.5% EN		12% unspecified panniculitis		10% EN 13% panniculitis
Nodules/papules/plaques	18.7% nodules 12.5% maculopapular	52% nodules 36% plaques	21.6% erythematosus papules				13% nodules 21% papules/plaques
Other	12.5% Sweet syndrome; 6.2% aspecific rash 6.2% fasciitis		8.6% urticaria		18% spongiotic dermatitis; 6% atopic dermatitis		10% urticaria
*Thromboembolism overall*	70%	44%	35.3%	30.77%	59%	NR	42%
DVT	64.2%	44%	NR	NR	DVT 35%	36%	26%
SVT	14.2%	NR	NR	NR	NR	14.6%	NR
PE	NR	4%	NR	NR	PE 18%	9%	11%
*Vasculitis manifestations overall*	NR	NR					
Aortitis and LVV			1.7%	NR	2%	2.2%	3%
Medium vessel vasculitis			NR	18%	NR	2.2%	4.4%
Small vessel vasculitis			NR	NR	NR	19%	14%
Ocular involvement overall	55% 54% scleritis/episcleritis 27.2% orbital cellulitis 18.2% orbital edema	28% 16% periorbital oedema 12% episcleritis 8% uveitis 4% scleritis 4% iritis	40.5 8.6% periorbital oedema	48.7% 33.3% periorbital oedema 9.5% uveitis 8.6% scleritis 12%episcleritis 3.4% orbital mass	59% 24% Orbital inflammation 12% scleritis 18% episcleritis 17.6% ocular venous thrombosis 17.6% anterior ischaemic optic neuropathy 12% anterior uveitis	51.7%	44% 20% periorbital oedema 15% orbital inflammation 11%episcleritis 10% scleritis 9% uveitis 5% conjunctivitis
Lung involvement	30% 83 % pleural effusion 16.6% NSIP 16.6% DAH	72% 72% infiltrates 32% pleural effusion	40.5% infiltrates 9.6% pleural effusion	41%	59% 23% organizing pneumonia 17.6% NSIP 12% usual interstitial pneumonia 12% nodules	NR	61% 46% infiltrates 12% pleural effusion 9% nodules 7% organizing pneumonia
PNS	15%	NR	7.7%	NR	12%	NR	5.1%
CNS	NR	NR	NR	NR	6%	NR	7.8%

CNS, central nervous system; DAH, diffuse alveolar hemorrhage; DVT, deep vein thrombosis; EN, erythema nodosum; LVV, large vessel vasculitis; MDS, myelodysplastic syndrome; MGUS, monoclonal gammopathy of uncertain significance; NR, not reported; NSIP, non-specific interstitial pneumonia; PE, pulmonary embolism; PNS, peripheral nervous system; SVT, superficial vein thrombosis.

Therefore, is important to avoid defining the disease solely based on the features described in the earliest reports, as this may overlook rarer or atypical manifestations. In this context, the present study takes a distinct rheumatological approach, positioning VEXAS syndrome within the broader spectrum of rheumatologic diseases. The observed differences compared to earlier cohorts may, therefore, reflect this specific focus, as seen in the variation of constitutional features.

An emerging challenge is how to quantify and assign appropriate weight to individual inflammatory manifestations. To address this and improve the classification of clinical features in patients with VEXAS syndrome, recent efforts have focused on developing a Disease Activity Index (DAI), as no validated scoring system for assessing disease activity currently exists. To date, the only proposed DAI for VEXAS syndrome is the VEXAS-CAF, which evaluates disease activity based on the presence or absence of 11 equally weighted manifestations (14). The VEXAS-DAI score was developed by an expert advisory committee using a modified Delphi methodology (5). This scoring system comprises 12 domains, with a total possible range of 0 to 40. Notably, hematologic manifestations are not included in the scoring, making the VEXAS-DAI primarily a clinical and inflammatory symptoms-based assessment tool. In our cohort, although limited to 20 subjects, the VEXAS-DAI was calculated for each patient, and a significant positive correlation was observed with the VAF% exhibited. This finding confirms and support that a higher proportion of cells carrying the *UBA1* mutation is associated with increased disease severity.

From a rheumatologic perspective, it is crucial to recognize also the aspect that many therapies commonly used in autoimmune and autoinflammatory diseases carry an inherent risk of infection. This is particularly relevant in our cohort, where all patients were managed within rheumatology settings and received immunomodulatory therapies. Notably, apart from one patient who received azacytidine in combination with prednisone, none of the patients were treated with conventional hematologic therapies. These findings emphasize the need for careful selection and monitoring of treatment, as both the underlying disease and the immunosuppressive therapies may contribute to a significantly increased risk of infections (15, 16). In our study, infections occurred primarily during treatment with corticosteroids and JAK inhibitors, pointing to a heightened risk of severe infections, including common seasonal and opportunistic pathogens. This underscores the importance of preventive strategies, such as vaccination and antimicrobial prophylaxis, in the management of VEXAS syndrome. Overall, these findings reinforce the central role rheumatologists play in both recognizing and managing the disease, particularly in patients with predominant inflammatory features.

## Conclusion

We collected a region-wide cohort of VEXAS patients, characterized by heterogeneous clinical manifestations, with a primary focus on the rheumatologic aspects of the disease. This study complements the longitudinal work of Gurnari et al., which provided an in-depth analysis of the hematological features of VEXAS in 41 Italian patients (17).

In this regard, a final consideration pertains to the issue of which specialist is most likely to encounter VEXAS patients initially. Although current estimates suggest that the prevalence of VEXAS syndrome in males ranges from 1 in 14,000 to as high as 1 in 4,000 in men over the age of 50 (18), our study, which identified 20 cases within rheumatological/internal medicine settings, yielded an estimated prevalence of approximately 1 in 70,000 among males over 50 in the Tri Veneto macro-region. While this likely reflects a substantial underestimation relative to current epidemiological data, it underscores a critical point: VEXAS syndrome remains markedly under-recognized, particularly outside of specialized or academic contexts. This discrepancy highlights the importance of a multidisciplinary diagnostic approach, involving rheumatologists, hematologists, dermatologists, and internists, to enhance early recognition and improve diagnostic accuracy for this complex multisystem disorder. For this reason, disease registries, such as those of the AutoInflammatory Disease Alliance (AIDA), will be indispensable for comprehensively capturing the full clinical spectrum of VEXAS syndrome (19).

Finally, a limitation of this study is the selection approach of subjects screened for genetic analysis, which, although highly specific, may have limited its sensitivity. Patients with milder or atypical phenotypes could have been excluded, possibly due to the influence of earlier literature that has shaped the disease definition. This narrow focus on initial descriptions might overlook a wider spectrum of clinical presentations, increasing the likelihood of missed diagnoses, particularly among individuals with less conventional symptoms.

## Data Availability

The original contributions presented in the study are included in the article/supplementary material. Further inquiries can be directed to the corresponding author.

## References

[1] Beck DB Ferrada MA Sikora KA Ombrello AK Collins JC Pei W . Somatic mutations in UBA1 and severe adult-onset autoinflammatory disease. N Engl J Med. (2020) 383:2628–38. doi: 10.1056/NEJMoa2026834, PMID: 33108101 PMC7847551

[2] Ferrada MA Savic S Cardona DO Collins JC Alessi H Gutierrez-Rodrigues F . Translation of cytoplasmic UBA1 contributes to VEXAS syndrome pathogenesis. Blood. (2022) 140:1496–506., PMID: 35793467 10.1182/blood.2022016985 PMC9523373

[3] Al-Hakim A Goldberg S Gaillard S Heiblig M Beck DB Savic S . Clinical features in VEXAS syndrome: a systematic review. Rheumatology. (2025) 64:5217–229. doi: 10.1093/rheumatology/keaf293/8164134, PMID: 40570089 PMC12494223

[4] ISTAT . Comunicato-territoriale/il-censimento-permanente-della-popolazione-in-veneto-anno-2021.

[5] Byram K Mann H Hammond D Savic S Kirino Y Gurnari C . Development of A disease activity index for the assessment of vexas syndrome (Vexas-dai). Ann Rheum Dis. (2025) 84:625–6. 10.1002/acr.80117 42439317

[6] Georgin-Lavialle S Terrier B Guedon AF Heiblig M Comont T Lazaro E . Further characterization of clinical and laboratory features in VEXAS syndrome: large-scale analysis of a multicentre case series of 116 French patients. Br J Dermatol. (2022) 186:564–74., PMID: 34632574 10.1111/bjd.20805

[7] Wolff L Caratsch L Lötscher F Seitz L Seitz P Coattrenec Y . VEXAS syndrome: a Swiss national retrospective cohort study. Swiss Med Wkly. (2024) 155:3879., PMID: 40132164 10.57187/s.3879

[8] García-Escudero P López-Gómez M López BM Dorta AG Frade-Sosa B Lizarzaburu MS . VEXAS syndrome through a rheumatologist’s lens: insights from a Spanish national cohort. Rheumatology. (2025) 64:3747–55., PMID: 39937690 10.1093/rheumatology/keaf094

[9] Zakine È Papageorgiou L Bourguiba R Mekinian A Terrier B Kosmider O . Clinical and pathological features of cutaneous manifestations in VEXAS syndrome: A multicenter retrospective study of 59 cases. J Am Acad Dermatol. (2023) 88:917–20., PMID: 36343774 10.1016/j.jaad.2022.10.052

[10] Sullivan MM Mead-Harvey C Sartori-Valinotti JC Kalantari K Kusne YN Patnaik MM . Vasculitis associated with VEXAS syndrome. Rheumatology. (2025) 64:3889–94. 10.1093/rheumatology/keae550 PMC12107069 39392442

[11] Borie R Debray MP Guedon AF Mekinian A Terriou L Lacombe V . Pleuropulmonary manifestations of vacuoles, E1 enzyme, X-linked, autoinflammatory, somatic (VEXAS) syndrome. Chest. (2023) 163:575–85., PMID: 36272567 10.1016/j.chest.2022.10.011

[12] Vitale A Caggiano V Martin-Nares E Frassi M Dagna L Hissaria P . Orbital/ocular inflammatory involvement in VEXAS syndrome: Data from the international AIDA network VEXAS registry. Semin Arthritis Rheum. (2024) 66:152430., PMID: 38554594 10.1016/j.semarthrit.2024.152430

[13] Muratore F Marvisi C Castrignanò P Nicoli D Farnetti E Bonanno O . VEXAS syndrome: A case series from a single-center cohort of italian patients with vasculitis. Arthritis Rheum. (2022) 74:665–70. doi: 10.1002/art.41992, PMID: 34611997 PMC8957507

[14] Kirino Y Maeda A Asano T Migita K Hidaka Y Ida H . Low remission rates and high incidence of adverse events in a prospective VEXAS syndrome registry. Rheumatology. (2025) 64:3872–8. doi: 10.1093/rheumatology/keae530, PMID: 39340799

[15] Vitale A Caggiano V Leone F Hinojosa-Azaola A Martín-Nares E Guaracha-Basañez GA . Efficacy and safety profile of biotechnological agents and Janus kinase inhibitors in VEXAS syndrome: data from the international AIDA Network VEXAS registry. Front Pharmacol. (2025) 16:1462254. doi: 10.3389/fphar.2025.1462254, PMID: 40046741 PMC11879931

[16] De Valence B Delaune M Nguyen Y Jachiet V Heiblig M Jean A . French VEXAS Group. Serious infections in patients with VEXAS syndrome: data from the French VEXAS registry. Ann Rheum Dis. (2024) 83:372–81. doi: 10.1136/ard-2023-224819, PMID: 38071510

[17] Gurnari C Pascale MR Vitale A Diral E Tomelleri A Galossi E . Diagnostic capabilities, clinical features, and longitudinal UBA1 clonal dynamics of a nationwide VEXAS cohort. Am J Hematol. (2024) 99:254–62. doi: 10.1002/ajh.27169, PMID: 38108611

[18] Beck BD Bodian DL Shah V Mirshahi UL Kim J Ding Y . Estimated prevalence and clinical manifestations of UBA1 variants associated with VEXAS syndrome in a clinical population. JAMA J Am Med Assoc. (2023) 24:318–24. doi: 10.1001/jama.2022.24836, PMID: 36692560 PMC10408261

[19] Vitale A Caggiano V Della Casa F Hernández-Rodríguez J Frassi M Monti S . Development and implementation of the AIDA international registry for patients with VEXAS syndrome. Front Med (Lausanne). (2022) 9:926500. doi: 10.3389/fmed.2022.926500, PMID: 35899212 PMC9309690

